# From Pesticide Pollution to Healthy Soils: Policy Assessment and Reform Options in Germany

**DOI:** 10.1007/s00267-026-02605-y

**Published:** 2026-11-03

**Authors:** Katharine Heyl, Jessica Stubenrauch, Felix Klickermann

**Affiliations:** Department of Environmental and Planning Law, Helmholtz-Centre for Environmental Research, Leipzig, Germany

**Keywords:** Pesticide policy, soil policy, EU policy, soil protection, soil pollution, soil health

## Abstract

Soil pollution in the EU and Germany is a pressing environmental issue. Multiple stressors contribute to soil pollution, including the use of plant protection products (here: pesticides). Policy goals, however, demand that the EU and Germany protect biodiversity and soils. Against this background, the present article adopts a qualitative governance analysis to investigate the extent to which German pesticide policies contribute to reduced pesticide pollution risks as well as biodiversity and soil health goals. Results indicate that (1) pesticide *use* requirements have a very limited steering effect on reducing pesticide pollution risks because they lack precision and enforceability, (2) pesticide *spatial* measures suffer from enforcement and control issues, an extensive exception regime and poorly implemented restrictions. (3) Overarching soil and nature conservation policies are largely ineffective as pesticide policies (lex specialis) override overarching policies. Still, certain policy elements such as training and certification, as well as the expansion of water protection areas contribute to some extent to reduced pesticide pollution risks, biodiversity and soil health. These results indicate that effective soil protection not only requires incremental changes in existing policies but comprehensive amendments to the agricultural sector including the adoption of economic instruments and measures from the global to the local level.

## Introduction

Healthy soils are a cornerstone of the biosphere and provide essential ecosystem services such as water filtration, nutrient cycling and carbon sequestration. As such, soil protection is directly linked to biodiversity (and climate) protection. Yet, soil health is endangered by multiple degradation processes such as erosion, acidification, compaction and pesticide pollution (Prăvălie et al. 2024). Pesticide pollution has been linked to negative impacts on soil biodiversity and consequently soil’s ability to provide ecosystem services. And whilst these developments have been identified at a global scale, they also affect soils in the EU and Germany (Tang et al. 2021; Beaumelle et al. 2023; Köninger et al. 2026).

To address these issues, countries around the world have committed to legally binding policy goals. The Convention on Biological Diversity for instance requires that biodiversity is conserved and its components – such as soils – sustainably used (United Nations 1992a). Legally non-binding global goals like the Sustainable Development Goals and the Kunming-Montreal Biodiversity Framework more precisely demand that by 2030, soil quality is progressively improved and pesticide pollution risks reduced (United Nations 2015; COP 2022). At the EU level, the Soil Monitoring Law aims at healthy soils by 2050 – a policy goal that is directly binding for all Member States including Germany (European Union 2025).

Against this backdrop, the present article aims to evaluate the steering effect of pesticide and nature conservation policies on pesticide pollution risks in soils and thus their contribution to legally binding biodiversity and soil health goals. To this end, a qualitative governance analysis is applied. The analysis focusses on Germany, as this policy research complements pesticide modeling activities conducted within SOILPROM in a use-case in Jülich in Germany. The analysis thereby builds on earlier work on pesticide policies at the EU level which found that current command and control policies are ineffective in reducing pesticide use and risks (Ekardt et al. 2024) and aims to answer the following research questions:

1. Which federal, state, district and local policies affect pesticide use and input into soils in the use-case area?
2. To what extent do these policies sufficiently reduce pesticide pollution risks and thus contribute to legally binding biodiversity and soil health goals?
3. How can existing policies be improved or supplemented or replaced with more effective policy instruments?

In analyzing pesticide policy, the present article engages in a long-standing discussion. Pesticide policy is comprehensively governed at the EU level but in some cases requires additional national policy. This is in particular true for pesticide use and spatial requirements where EU policy establishes an overarching framework that demands completion at the national or state level (e.g., Directive 2009/128/EC). In line with that, pesticide policy research has primarily focused on the EU level. For instance, Bozzini (2017) comprehensively analyses the EU policy framework and identifies enforcement and data issues. Others point out that EU pesticide policies fail to reduce pesticide use and risks and simultaneously prioritize short-term economic interests above long-term environmental health (Garcia-Caro 2023). Within the EU policy framework, studies have frequently focused on the assessment of active substances. Researchers at the European Food Safety Authority published a detailed overview of the risk assessment of Neonicotinoids (Auteri et al. 2017) – a risk assessment procedure which has often been criticized by NGOs and researchers alike (Sgolastra et al. 2020; Brühl and Zaller 2022; Pesticide Action Network Europe 2023; European Academies Science Advisory Council 2025). Criticism includes, for instance, a lack of compliance and transparency mechanisms (Storck et al. 2017; Mie and Rudén 2022) and insufficient assessment of pesticide mixtures (Panizzi et al. 2017; Brühl and Zaller 2019). Another issue that is frequently discussed is the role of Member States in the market approval of pesticides. De Braal (2023) for instance finds that Member States are very differently involved in the approval process which could negatively influence the risk assessment of an active substance. Others point out that national derogations from EU standards limit the effectiveness of EU pesticide policy (Bozzini 2017; Alonso González et al. 2021).

Hence, much attention has so far been targeted at the market approval and risk assessment processes at EU level but some studies have also focused on pesticide policies in EU Member States such as Spain and Denmark (Pedersen and Nielsen 2017; Alonso González et al. 2021). However, Germany has not been at the center of these analyses (Lee et al. 2019, p. 4) except for some dated studies (Bürger et al. 2008; Barzman and Dachbrodt-Saaydeh 2011) and studies that have been published in German (Borwieck 2017; Möckel et al. 2021a; Douhaire 2022).

The following sections outline the method, present the results and develop policy proposals which then lead into the discussion and conclusions.

## Method

### Conceptual Background

A qualitative governance analysis is applied to assess the steering effect of German pesticide and nature conservation policies on pesticide pollution risks in soils and thus their contribution to legally binding biodiversity and soil health goals. In order to cover all policy levels from the federal to the local level, a use-case area serves as reference area for state, district and local policies.

To effectively assess policies, the underlying context needs to be understood. The local context of this qualitative governance analysis is a use-case in Germany. In the area, land is predominantely used for agriculture and pesticides are applied to 84% of agricultural land with pesticide intensity^1^ being frequently higher compared to other regions in Germany (Baum et al. 2025, pp. 192–193; Federal and State Statistical Offices [editor] 2025). As such, the use-case is principally subject to pesticide pollution risks. Administration-wise, the use-case is located in the state North Rhine Westphalia (NRW) and the district Düren and covers land of two municipalities (Jülich and Niederzier). The state policies of NRW, district policies from Düren and local policies from Jülich and Niederzier henceforth apply to the use-case area – alongside federal and EU measures.

In order to evaluate the contribution of pesticide and nature conservation policies to legally binding policy goals, the relevant policy goals have to be identified. As the impacts of pesticides on soils primarily affect biodiversity and soil health, the goals of the Convention on Biological Diversity and EU Soil Monitoring Law are useful policy goals for the present analysis. *Hence*, o*verall, this qualitative governance analysis assesses the extent to which the investigated policies contribute sufficiently to reduced pesticide pollution risk in the use-case area and thus their contribution to the goals of the Convention on Biological Diversity and EU Soil Monitoring Law*.

The steering effect of these policies on reducing pesticide pollution risks serves to estimate their contribution to the overarching goals.  Pesticide pollution risk depends on multiple intertwined factors including pesticide exposure and effects, ecological and landscape context as well as (soil) management practices (Schäfer et al. 2019). Against this background, pesticide pollution risks have been estimated for human health (Maggi et al. 2021; Galimberti et al. 2025), non-target organisms in water bodies (Herrero-Hernández et al. 2020) and soils (Froger et al. 2023). Usually, these estimations include a pesticide quantity component and a toxicity component and some policy goals likewise incorporate this differentiation. The EU for instance aims to reduce the overall use (quantity) and risk (toxicity) of chemical pesticides by 50% by 2030 (European Commission 2020). The present analysis therefore qualitatively investigates if selected policies contain measures which target pesticide pollution risks quantitatively and toxicity-wise.

In addition to this angle, the qualitative governance analysis looks into typical governance problems (Table 1). The (non-)presence of governance problems indicates if a policy instruments effectively contributes to a policy goal or if these problems hinder that. For instance, economic policy instruments which only target pesticide quantity might induce shifting effects to less but more toxic pesticides which limits or even worsens the steering effect of these policies on pesticide pollution risks in the environment (Nielsen et al. 2023; Finger and Pedersen 2025). Likewise, enforcement problems of pesticide spatial measures likely contribute to increased pesticide pollution in water bodies – thus undermining the effectiveness of these measures as well (Federal Office for Consumer Protection and Food Safety 2026). Having said that, not all policies suffer from all governance problems and some governance problems overlap. For instance, shifting effects can make policy enforcement more challenging because pesticide pollution might fall into the responsibility of several public authorities in different locations.

**Table 1:** Typical governance problems (based on Stubenrauch and Heyl (2026))

Governance problem	Description
Lacking target stringency	Target stringency describes the ability of a policy instrument to achieve a policy goal. Policy elements such as voluntariness or bindingness, scope and instrument choice affect the ability to achieve a policy goal. An assessment of EU soil policies for instance concludes that these policies lack effective measures to protect soils because they heavily rely on voluntary measures (Heuser 2022) and an overall accelerated progress towards soil protection policy objectives is needed (European Commission 2025a). Lacking target stringency impedes achieving a policy goal.
Lacking legal clarity	Legal clarity means that provisions are precise so that specific and enforceable requirements for addressees can be derived. Lacking legal clarity instead creates uncertainties and ambiguities. A prominent example in EU environmental policy are not or inadequately defined legal terms such as e.g., “environmental damage” and “local population” (Pingen 2012; de Smedt and Gerritsen 2024).
Problem of measurement and monitoring (“complexity issue”)	A problem of measurement and monitoring describes difficulties in accurately mapping, monitoring or measuring specific (environmental) phenomena, despite the relevance of this information for policies (see e.g., Cato 2020, chap. 8.3). Alblas et al. (2025) for instance discuss the issue that soil policy requires scientific certainty but that lacking knowledge on e.g., soil threats and management hinders effective policy design.
Rebound effects	Rebound effects describe situations where the efficiency of a process is improved while overall resource use increases simultaneously. Typical examples for rebound effects in agriculture is increased land use and irrigation due to efficiency gains (Paul et al. 2019).
Shifting effects (“leakage”)	Shifting effects transfer (environmental) problems from one region/country/sector/ product to another. Möhring et al. (2019) point out that pesticide policies which rely on quantitative reduction targets may induce pesticide users to shift to more toxic pesticides.
Enforcement and control problems	Enforcement and control mechanisms ensure that a policy is correctly implemented and complied with. Problems of enforcement and control hinder that. Enforcement and control of German pesticide policies reveals that, in 2025, pesticide users were in breach with spatial measures in 33% of the controls (Federal Office for Consumer Protection and Food Safety 2026).

### Qualitative Governance Analysis–Procedure

In a first step, all potentially relevant policies have to be identified and those which will be subject to the analysis selected. To this end, a broad policy search was conducted and preliminary scoping results from SOILPROM used (González Alcaraz and Aguilar Garrido 2025, pp. 137–147). The website from the Federal Ministry of Agriculture, Food and Regional Identity (Federal Ministry of Agriculture, Food and Regional Identity 2025a), the Chamber of Agriculture in NRW (Chamber of Agriculture NRW 2026) and the official law databases of the federal (Federal Ministry of Justice 2026) and state level (NRW Interior Ministry 2026) served as “database” for the policy scoping. Besides that, in May and June 2025, the state environment and agricultural ministry, the district environment authority and the cities of Jülich and Niederzier were contacted to request the latest developments on pesticide policies.

**Fig. 1:**
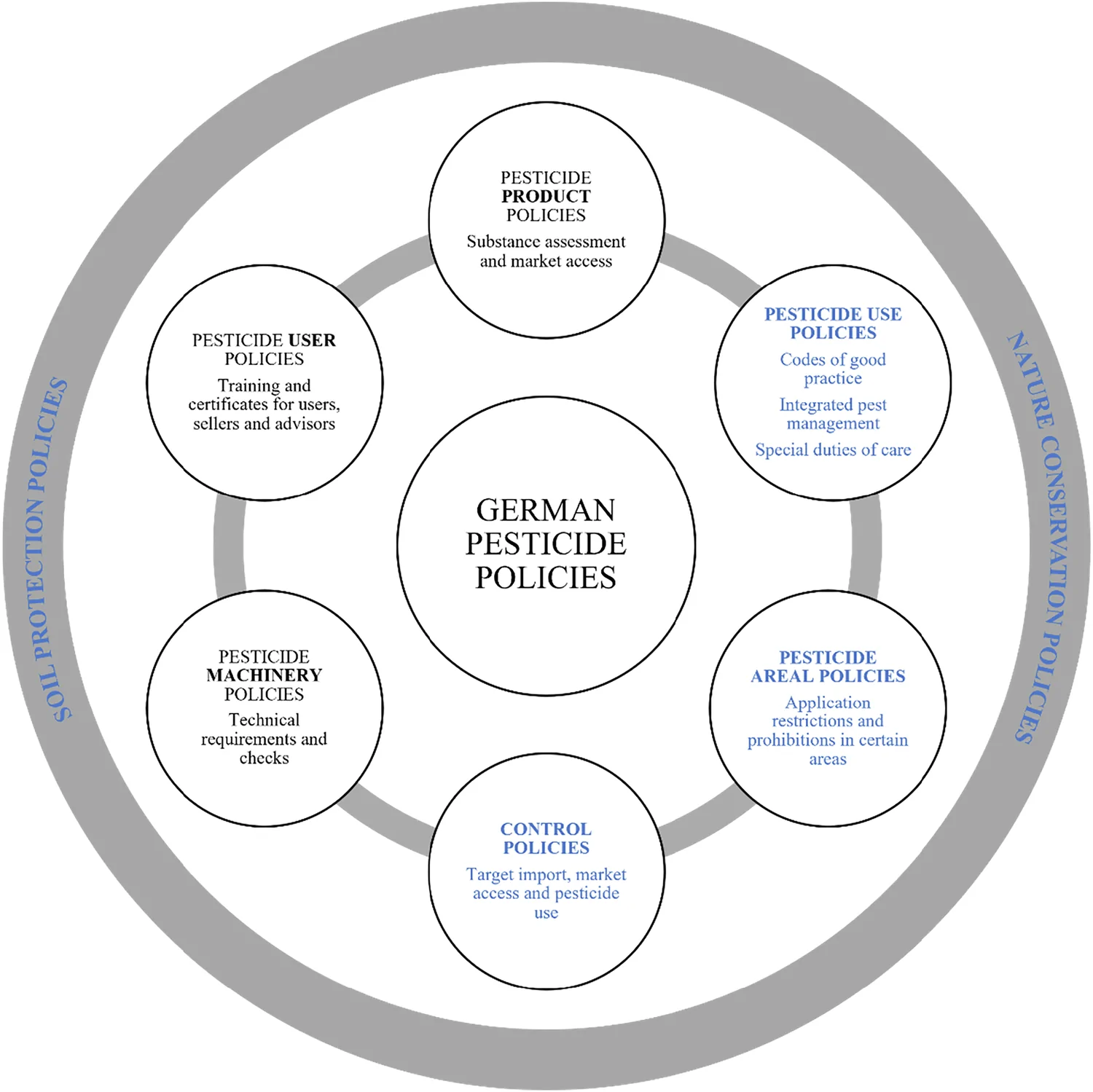
Overview of German pesticide policies and selected policies for the governance analysis in blue (own figure based on Federal Ministry of Food and Agriculture (2013) and Pingen (2012)

The scoping revealed a long list of potentially relevant policies which needed to be narrowed down to keep the analysis feasible. To this end, the policies were categorized by content. (1) Pesticide USE policies (section “Pesticide Use Policies”), (2) pesticide SPATIAL policies (section “Pesticide Spatial Policies”), (3) soil protection policies (section “Soil Protection and Nature Conservation Policies”), (4) nature conservation policies (section “Soil Protection and Nature Conservation Policies”) and (5) the corresponding control policies at the federal, state, district and local level were chosen for this analysis because they are most applicable to the use-case area (Fig. 1, in blue). In addition to these “direct” pesticide policies, other policies from e.g., water protection, chemicals and agricultural policy also target pesticides. However, it is beyond the scope of this article to assess these policies ﻿– although occasional references to agricultural and chemicals policy will be established in the discussion.

In a second step, the legal texts of the policies were subjected to a qualitative text analysis which contains elements of a legal analysis. As such, the policies are interpreted in particular systematically, teleologically and historically ﻿– meaning in relation to other policies, purpose and evolution (Ammann 2020). Policies are then investigated with a view to their steering effect on pesticide pollution risks (quantity and toxicity) and potential governance problems.

In the last step, based on the assessment results, alternative policy instruments or policy amendments are developed. These proposals aim to circumvent the governance problems of the existing policies and to comprehensively address the underlying environmental issue. The recommendations in this article build on and extend earlier research on climate change (Ekardt et al. 2022), biodiversity (Weishaupt et al. 2020; Ekardt et al. 2024), phosphorus (Heyl et al. 2023; Garske et al. 2025), plastics (Stubenrauch and Heyl 2026) and forest policy research (Stubenrauch and Garske 2023).

## Results

### Pesticide Use Policies

Pesticide use policies are scattered across several federal laws, state laws, ordinances, administrative regulations, enactments^2^ and non-binding documents (Table 2). The state NRW does not establish any specific pesticide use requirements for the use-case area – despite having the legal leeway to do so. Likewise, the soil protection plan of the district Düren does not explicitly refer to or map pesticide contamination of soils (District Düren 2005).

**Table 2:** Pesticide use policies

	Topic	Content	Source
General requirements	Market approval	Market approval is the precondition for any pesticide use. Pesticides must only be used as established in the approval. The approval may include e.g., maximum applications per year and maximum dose per hectare. Exceptions are established for e.g., emergency situations.	§ 12 PflSchG
	Personal qualification*	A training certificate is required to use plant protection products professionally. This includes farmers.	§ 9 PflSchG & § 6 State Ordinance on the implementation of the PflSchG
Use requirements	Health and environmental restriction*	Pesticides must not be used if they cause health, animal or water damage. Pesticides must not impact wild animals and their habitats. Exceptions are established to e.g., prevent substantial damage to agricultural production and local vegetation and animals.	§ 13 PflSchG
	General use restrictions	A complete or limited ban is established for different pesticides as well as use restrictions. Use restrictions for glyphosate are established and exceptions for research.	§§ 1-3b, 7 PflSchAnwV
	Environmental restriction	Pesticides which have been classified as dangerous for bees must not be applied on flowering plants and plants which are visited by bees. Exceptions are established for e.g., significant plant damage and research.	§§ 2-3 BienSchV
	Aerial use restriction	Aerial use is prohibited without a permit. A permit can be issued for pesticide use in wineries and forests.	§ 18 PflSchG
		The aerial use permit establishes e.g., use areas and time, suitable climatic conditions and risk reduction measures. It is temporarily granted.	§ 2 PflSchMAnwLuftFzgV
	Seeds and plants use restriction	Seeds and plants which contain pesticides must only be used if the pesticide is approved for the market. Use is prohibited if it causes health, animal or environmental damage.	§ 19 PflSchG
	Maize seed restriction	Maize seeds which have been treated with certain pesticides must not be sown or sown with precautionary measures. Exceptions for e.g., experimental purposes are established.	§§ 3, 4 MaisPflSchMV
Use obligation	Phylloxera management	Vine owners are required to inform a public authority if they observe or suspect an infestation with phylloxera. They have to manage the pest if requested by the authority including by monitoring and removing vines. If infested, moving vine materials and planting is restricted.	§§ 1–4 ReblV
	Potato ring rot and brown rot management	Potato and tomato producers are required to inform a public authority if they observe or suspect an infestation with potato ring rot and brown rot. If infestation is confirmed, the producers have to either destroy or separately handle potatoes and tomatoes. Planting these crops is restricted or limited and machinery cleaning required.	§§ 1, 3, 6-11 KartRingfV
	Use obligation (fruits)	Owners of fruit plants have to manage specific pests if requested by a public authority. Pests include e.g., fireblight and apple proliferation.	§§ 2,5 PflBestSchV
Use principles	General use principles*	Plant protection may only be done in line with good professional practice. Good professional practice includes (1) the EU principles on integrated pest management, (2) preventive measures, avoidance of the introduction and spread of harmful organisms, control and management of harmful organisms and support of natural pest management measures, (3) measures to avoid or protect against the health and environmental risks from pesticide use.	§3 PflSchG
	Good professional practice*	The good professional practice incorporates integrated pest management. It aims to reduce chemical plant protection by prioritizing physical and non-chemical methods. General principles and specific principles are established.	Good professional practice document
End of life	Disposal obligation	Pesticides without valid approval or which have been banned must be disposed of in line with waste and circular economy law.	§ 15 PflSchG
Control and sanctions*	State obligations	States are responsible for the implementation and control of the Plant Protection Law and its ordinances.	§ 59 PflSchG
	Offences and penalties	Breaching obligations is punished with fines or imprisonment depending on the case (“mild breach” – “Ordnungswidrigkeit”; “hard breach” – “Straftat”).	§§ 68 - 69 PflSchG
	Offences	Using pesticides in contravention with the use obligations is a mild breach.	§ 4 BienSchV
	Offences	Violating an order issued by a public authority (“Anordnung”) is a mild breach.	§ 6 MaisPflSchMV
	Offences	Applying a prohibited pesticide is a hard breach and violating against use restrictions a mild breach.	§ 8 PflSchAnwV
	Offences	Mild breaches are defined for several cases including failing to report infestation and destroying potatoes and tomatoes.	§ 14 KartRingfV
	Offences	Mild breaches are defined for multiple cases including failing to report infestation and transporting vine materials into non-infested municipalities.	§ 7 ReblV
	Offences	Not following an order by a public authority constitutes a mild breach.	§ 7 PflBestSchV

Pesticide use policies can be distinguished into (1) general requirements such as market approval and personal qualifications, (2) use restrictions including environmental and health restrictions, (3) use obligations to manage specific pests, (4) use principles such as good professional practice guidelines, (5) end of life obligations and (6) control and sanctions. All of these instruments have in common that they are command and control instruments or voluntary measures. They thus prescribe, restrict or ban certain actions either on a mandatory or voluntary basis. Below, central provisions are assessed (Table 2, marked with *).

### Policy Assessment

The good professional practice should arguably be a key element of pesticide use policy and in fact, Pingen (2012) calls these practices “basis strategy”. The good professional practice contains measures which can contribute to reduced pesticide quantity and toxicity for instance by giving priority to non-chemical plant protection measures and limiting pesticide doses and frequency to levels that are necessary. However, the practices have been criticized for being too vague ﻿– despite being 52 pages long and being more precise than the good professional practice guidelines in soil and nature conservation law (section “Federal and State Soil Protection Law”; Pitschel 2021; Koch et al. 2024). The good professional practice furthermore suffers from obsolescence. The Plant Protection Law requires policy makers to incorporate the latest scientific findings into the good professional practice. However, the practices have not been updated since 2010. As such, research results which have since 2010 reduced knowledge gaps on pesticide pollution in soils are thus not incorporated into the practices. In addition to that, neither the good professional practices nor the general use principles are directly sanctionable obligations. Sanctions only apply if a public authority issued an order to perform certain actions (Pingen 2012; Rehbinder 2018; Pitschel 2021; Köpl and Beck 2022). Hence, *control and enforcement* are virtually impossible unless a public authority has requested certain actions (“Anordnung”). Overall, the steering effect of the good professional practice and the general use principles to reduced pesticide pollution risks in the use-case area is very limited as is their alignment with biodiversity and soil health goals.

Aside from these two measures, the PflSchG establishes an overarching pesticide use restriction if environmental and health risks seem likely (§ 13 PflSchG). While at first sight this restriction appears useful to reduce pesticide pollution risks (quantity and toxicity) and to contribute to biodiversity and soil health goals, the PflSchG also establishes that the restriction does not apply to plant protection measures which are applied in line with the good professional practices.^3^ However, given the identified shortcomings of the good professional practices above, this overarching restriction does not induce behavior change and *lacks target stringency*. Having said that, public authorities are empowered to on the one hand adopt more stringent measures and on the other hand to adopt further exceptions to e.g., avoid substantial agricultural damages. While this flexibility may enable localized interventions, it also dilutes the restriction and in doing so potentially undermines its *target stringency* and *legal clarity* as pesticide use obligations can vary from one location to another. Legal clarity is also lacking because of not-defined legal terms. For example, the Plant Protection Law prohibits disturbing the “local population” of protected species during pesticide application. Yet, it is unclear what a “local population” is as a legal definition is lacking (Pingen 2012). Overall, these governance problems limit the contribution of this measure to biodiversity and soil health goals and its steering effect on reducing pesticide pollution risks in the use-case area appears bounded.

Another instrument that could induce reduced pesticide pollution risks quantity and toxicity-wise are training and advisory services. In fact, research frequently points out that these tools are needed to better manage pesticide use and risks (Damalas and Koutroubas 2017; Lee et al. 2019; Möhring et al. 2020; Mance et al. 2025). Hence, the legal obligation to obtain a training certificate seems generally useful to induce reduced pesticide quantity and toxicity risks. The certificate requires user knowledge about plant protection products, application and machinery as well as the overarching principles on the sustainable use of plant protection products. As such, while knowledge about pesticide pollution risks is certainly needed, an even stronger weight could be set on e.g., integrated pest management. The certification scheme thus has a limited steering effect to reduce pesticide pollution risks in the use-case area.

Lastly, command and control instruments require effective control and enforcement mechanisms. To this end, the PflSchG consolidates offences and penalties of several pesticide policies. It lists multiple cases which are fined with either up to 50,000 EUR or 10,000 EUR. Imprisonment is established for breaches against market approval and protected species. Outside the scope of this Law, ordinances do not establish further penalties which means that the general penalty law applies with fines up to 1000 EUR. In 2023, only 1.5% of the agricultural businesses in Germany were subject to a control and 21% of the controls revealed a violation against pesticide use measures (Federal Office for Consumer Protection and Food Safety 2025b). The Federal Office for Consumer Protection and Food Safety points out that the controls are done “risk-oriented” but these numbers nevertheless reveal *enforcement issues –* and substantial administrative burdens.

### Pesticide Spatial Policies

This section examines German pesticide spatial policies with a focus on NRW as well as challenges related to enforcement and regulatory effectiveness.

#### Areas used for “agricultural, forestry or horticultural purposes” and “surface waters”

The authorization of pesticides contains not only pesticide use requirements, but also defines application areas. Accordingly, pesticides may only be used in the areas listed in the authorization (§ 12 (2) No. 1 PflSchG). Such areas are typically areas used for agricultural, forestry or horticultural purposes. Using pesticides on paved open areas or areas for other purposes is generally prohibited. Furthermore, pesticides must not be applied in or directly adjacent to surface waters or coastal waters (§ 12 (2) PflSchG). In NRW, these rules have been concretized by an administrative regulation (Ministry for Environment, Spatial Planning and Agriculture NRW 2000). The Regulation establishes that for land to have “agricultural, forestry or horticultural purposes”, permanent land management that aims to produce plants or plant products, or land management for horticultural design and maintenance is needed. The decisive factor is the actual use. Areas that serve agricultural or horticultural purposes indirectly such as paths, roadside greenery and hedges are excluded from the scope of this term. Besides that, when applying pesticides, a minimum distance of 5 to 10 meters must be maintained from the top of the bank of surface waters (§ 4a (1) PflSchAnwV).

There are two significant exceptions to this area-based rule: Firstly, in certain situations, the competent authority can allow pesticide use on paved outdoor areas that are not used for agricultural, forestry or horticultural purposes (§ 12 (2) PflSchG). This exception requires (1) an urgent purpose of high importance, (2) a lack of alternatives at reasonable costs and (3) absence of contrary overriding public interests (esp. human and animal health or nature protection). One example for such an exception was a general order between 2021 and 2025 which permitted the use of pesticides to control giant hogweed in roadside greenery. The exception was justified on the grounds of severe skin reactions upon plant contact (Chamber of Agriculture in North Rhine Westphalia 2021). Secondly, the competent authority can allow pesticide use for areas that are not listed in the authorization if the pesticide application is intended for plants cultivated on a small scale or against pests that cause significant damage only in certain areas (§ 12 (6) PflSchG). The Chamber of Agriculture in NRW makes use of this exception. It has issued lists of certain pesticides and how they can be used to manage pests in e.g., tree nurseries (Chamber of Agriculture in North Rhine Westphalia 2024).

Besides that, *how* pesticides are applied can also be relevant for the application area. For example, authorizations for pesticide application by aircraft should only be granted to control pests in vineyards on steep slopes and in forest canopies. Aircraft application demands that protective requirements for residential areas and others are met (§ 18 PflSchG) in order to exclude and minimize “pesticide drift” (Metzger 2026). Protective requirements include safety distances, specific seasonal and weather conditions, the application of the best available technology for minimizing drift, and the provision of timely information to local residents and other people who may be in the immediate vicinity of the application areas (§ 2 (1) PflSchMAnwLuftFzgV). Lastly, the good professional practice for plant protection contains further rules which partly cover spatial measures (section “Pesticide Upatial Policies”).

#### Nature Conservation Areas

In Germany, pesticide use is mostly prohibited in nature conservation areas, national parks, natural monuments and biotopes (§ 4 (1) PflSchAnwV). Pesticide use is also prohibited in certain areas of common importance (§ 7 (1) No. 6 BNatSchG, § 4 (1) S. 2 and (3) PflSchAnwV). Besides that, in NRW, voluntary agreements and exceptions aim to eliminate pesticide application in certain types of arable and agricultural land. However, a pesticide-free management in these areas has not been achieved by the targeted deadline in 2024 (Federal Ministry of Food and Agriculture 2024).

Nevertheless, NRW has adopted some pesticide spatial measures for nature conservation areas:

1. using pesticides on permanent grassland in nature conservation areas has been prohibited since 2022 ﻿– albeit with a few exceptions (§ 4 (1) S. 1 Nr. 6, (2) S. 4 LNatSchG NRW);
2. NRW provides subsidies to compensate additional expenses which results from special restrictions (Ministry of Agriculture and Consumer Protection 2023) and
3. the total nature conservation area has grown significantly over the past few decades (State Agency for Nature, Environment and Climate NRW 2025) and two of these areas are located in the use-case area (“NSG Lindenberger Wald” and “NSG Burgauer Wald”).

Still, competent authorities may allow the use of certain pesticides in nature conservation areas in order to (1) prevent significant agricultural, forestry or other economic damage; (2) protect native flora and fauna particularly from invasive species and to (3) ensure the safety of railway lines. To this end, a decree establishes an economic threshold (*6%-criteria*) which determines whether “significant economic damage” has occurred (Chamber of Agriculture in North Rhine Westphalia 2026). The District Düren applies this exception. It establishes that in the “NSG Lindenberger Wald”, pesticides can exceptionally be used to address forestry calamities and to prevent the spread of invasive plant species (District Düren 2024, p. 66).

#### Water Protection Areas and Landscape Plans

The state government in NRW may adopt orders to designate water protection areas if public welfare requires the prevention of erosion and pesticide inputs into water bodies (§ 51 (1) Nr. 3 WHG, §§ 35 (1), 112 ff. LWG NRW, § 1 (3) and Annex II No. 20.125 ZustVU). In these areas, restrictions on the use and handling of pesticides are determined on a case-by-case basis. One such water protection area is established in the use-case area. The “WSG Titz” is divided into different zones. While in Zone I, the use of pesticides is prohibited, in Zone III, storing pesticides requires a special permission (District Goverment of Cologne 1977). Besides that, further water protection areas are currently designated in Jülich and Düren including the “WSG Koslar-Barmen” and the “WSG Niederzier-Ellen”.

### Policy Assessment

Pesticide spatial measures in NRW such as the expansion of water protection areas (which is also required due to the increasing issue of water scarcity), the extension and increased protection of nature conservation areas and the provision of compensatory subsidies demonstrate that the current policy framework to some extent contributes to reducing pesticide pollution risks (quantity) in the use-case area.

However, the analysis also reveals a number of governance problems. Pesticide spatial measures are highly context-specific and therefore contain small-scale and detailed rules which easily cause *enforcement and control problems*. And in fact, the Federal Office for Consumer Protection and Food Safety has identified a significant number of violations of (1) the minimum distance requirement for water bodies, (2) the rule for using pesticides only on designated areas, and (3) the prohibition to use pesticides on paved or open areas (Federal Office for Consumer Protection and Food Safety 2025a, b). In 2025, official controls found that one third of the inspected farms (i.e., 230 out of 687) had violated (1) the ban on the application of plant protection products near water bodies (§ 4a PflSchAnwV) taking into account the relevant provisions of state law, as well as (2) compliance with product-specific application provisions (Federal Office for Consumer Protection and Food Safety 2026, p. 49). (Federal Office for Consumer Protection and Food Safety 2025a, p. 32). Further exaggerating this issue is the comprehensive exception regime. However, lacking transparency regarding the exeptions granted in NRW makes a comprehensive assessment difficult. The particular environmental relevance and the need to evaluate the practice thus call for an easier access to information. Still, as discussed above, exception regimes increase regulatory complexity and impede *enforceability and transparency*. It could also lead to an increased quantitative use and risk of pesticides on areas which are not subject to spatial restrictions and thereby cause *shifting effects*. Overall, these governance problems substantially reduce the potential steering effect of pesticide spatial measures and with that their contribution to sufficiently reduce pesticide pollution risks in the use-case area.

### Soil Protection and Nature Conservation Policies

This section analyses overarching policies on soil and nature conservation.

#### Federal and State Soil Protection Law

The German Federal Soil Protection Law (BBodSchG) aims to sustainably secure or restore soil functions (§ 1 BBodSchG) and, in accordance with § 7 BBodSchG, to take precautionary measures to prevent harmful changes to the soil in line with the “precautionary principle” (United Nations 1992b). However, the BBodSchG is subordinate to special laws (*lex specialis*), such as pesticide laws (§ 3(1) Nos. 1–11 BBodSchG). It thus only applies where these special laws do not regulate the effects on the soil (principle of subsidiarity).

According to the BBodSchG, precautionary soil protection has to be ensured through “good professional practice” in agriculture (§ 17 BBodSchG). “Good professional practice” requires that soil cultivation is site-adapted and takes weather conditions into account so that soil fertility and productivity are protected. The soil structure has to be preserved and improved, natural structural elements maintained, soil compaction and erosion avoided and the biological activity of soil and the humus content preserved. The latter may be achieved by reducing the cultivation intensity (17 (2) no. 7 BBodschG), which might, in case of regenerative agricultural practices, reduce pesticide use as well (Pagana et al. 2025; Congreves 2025). Notably, these principles are conveyed only through advisory services and are thus not legally binding (Möckel 2020, p. 6). Additionally, in contrast to precautionary obligations outside agricultural land use (§ 10 BBodSchG), § 17 BBodSchG lacks a mechanism to enforce the good professional practice.

States are largely responsible for enforcing the BBodSchG. To this end, NRW adopted the State Soil Protection Law of NRW (LBodSchG). The Law once again requires precautionary soil protection including by preventive measures against harmful soil changes, particularly through avoiding the introduction of harmful substances (§ 1 (2) No. 1 LBodSchG). It does not, however, further specify the good professional practice requirements of the federal BBodschG. Therefore, the above measures apply.

### Policy Assessment

The soil protection legislation related to healthy (agricultural) soils basically suffers from three governance problems: lacking target stringency, lacking legal clarity and enforcement deficits, which are highly intertwined and reinforce each other.

*Lacking target stringency* constitutes first and foremost in the fact that soil protection in line with the precautionary principle cannot take place on agricultural land due to the principle of subsidiarity. The principle of subsidiarity leads to the unfortunate situation that the primacy of *lex specialis* over the overarching soil protection law applies even if soil protection measures in the special laws fall short of the BBodSchG standard (Bodle et al. 2026). And in fact, as the policy assessment on pesticide use policies ﻿– the *lex specialis* – reveals (section “Pesticide Use Policies”), precautionary soil protection is not established there.

*Lacking legal clarity* is the second major governance issue impeding the alignment of soil protection policies with biodiversity and soil health goals. The principles of good professional practice remain vague and are neither concretized in the Federal Soil Protection Ordinance (Möckel et al. 2014) nor State Law of NRW. Thus, the concrete meaning of the good professional practice can also not be further defined by courts and legally binding measures to e.g., curb pesticide use and risks (quantity and toxicity) at certain locations for preventive soil protection cannot be imposed and only non-binding advice be offered.

Good professional practice can therefore neither be controlled nor enforced by authorities, leading scholars to call them hollow “legal rhetoric” (Möckel 2020, p. 5), and causing the third governance problem: *lacking enforceability*. In fact, the legally non-binding character combined with missing enforcement mechanisms privilege agricultural land use compared to other land use types (Ginzky 2024). All this stands in sharp contrast to the crucial role of agricultural land use for soil health (Vieira et al. 2024; Köninger et al. 2026). Thus, despite aiming at precautionary action, German soil protection policies fail to implement the “precautionary principle” and do not reduce pesticide pollution risks (quantity and toxicity) in the use-case area. Hence, the measures of the soil protection policies seem inadequate to sufficiently contribute to biodiversity and soil health goals. Yet, despite these shortcomings, attempts to reform soil policies and align them more closely with the “precautionary principle” have failed so far.

#### EU Soil Monitoring and Resilience Directive

The EU Soil Monitoring and Resilience Directive (Soil Monitoring Law, SML) is the first overarching EU soil protection legislation in EU history, which has to be implemented in the Member States’ national law, such as in Germany. The SML establishes a coherent soil monitoring framework for all soils across the EU. It furthermore aims to reduce soil contamination to levels that are no longer considered harmful to human health or the environment, and to continuously improve soil health. The overarching goal is to achieve healthy soils by 2050. All aspects of soil degradation shall therefore be addressed, and healthy soils shall be maintained in healthy conditions (Art. 1 SML).

The monitoring framework incorporates multiple soil descriptors and soil health criteria. These criteria include “non-binding sustainable target values” which cover good soil conditions, and “operational trigger values” which indicate poor soil conditions (Art. 7(2) SML). Annex I SML specifies the soil descriptors and related criteria. While criteria for healthy soil conditions shall be established at Union level for all soil descriptors listed in Part A of Annex I, Member States are responsible to define those for soil descriptors listed in Part B. No criteria have to be defined for soil descriptors listed in Part C.

“Concentration of selected active substances in pesticides and their metabolites” is a soil descriptor listed in Annex 1 Part C. This categorization into Part C has far-reaching consequences:

1. As neither non-binding sustainable target values nor operational trigger values have to be defined, only specific administrative and operational tasks arise for Member States: They are required to establish a national list of specific contaminants that, due to their toxicity, persistence and mobility, pose the highest risk to human health and the environment within their territory (Art. 7(4) SML). This list shall consider an “indicative list of soil contaminants” with potential significant risks to soil health and soil resilience co-developed by the European Commission and the Member States (Art. 8 SML).
2. Sampling points for pesticides may be limited to a “relevant subset” of the total number of sampling points required under Art. 9 SML and therefore do not need to be as comprehensive as those for other soil descriptors. Besides that, unified methodologies are completely lacking, meaning that results on stressor-related soil biodiversity loss, i.e., due to pesticide inputs, might not be comparable between Member States. Aside from that, the data collected must be submitted electronically to the Commission and the European Environment Agency (EEA) every six years, contributing to the “indicative list of soil contaminants” and helping to identify Union-wide contamination patterns (Art. 8(1), Art. 19(1) SML). To ensure the long-term preservation of these findings, representative subsets of soil samples must be stored in specialised physical soil archives for at least two monitoring cycles (see Art. 9(11) SML). This facilitates the re-evaluation of samples for subsequent medical or ecological research, or policy evaluations, and enables the comprehension of long-term trends in soil contamination by pesticides.
3. The Directive imposes a qualitative analysis requirement on Member States: It is their responsibility to determine whether the measured contamination levels lead to a critical loss of ecosystem services (Art. 10(3) SML).

Notably, while the obligations described above create an evidence base, even pesticide pollution values that pose a significant risk to soil health and soil resilience do not automatically trigger an obligation to support landowners (Art. 11 SML) or to remediate the site in the general monitoring process. In fact, a direct, operational remediation obligation arises only when these substances constitute an “unacceptable risk” (Art. 16(2) SML) within the framework of a site-specific risk assessment for contaminated sites ﻿– again under the responsibility of the Member States (Art. 13, Art. 16(4) SML). Only then is the “polluter-pays-principle” potentially applied by the Member States (recital 47 SML). Thus, the findings from soil monitoring are not incorporated into pesticide approval procedures and soil remediation measures, and feedback loops between monitoring results and authorisation procedures are missing thus far (Kotschik et al. 2024). The steering effect does therefore not extend beyond a data-driven early-warning system.

Aside from the soil descriptor discussed above, the loss of soil biodiversity, which can be increased by pesticide application (Hallmann et al. 2017), is also categorized under Annex I, Part C SML. DNA metabarcoding, i.e., the identification of multiple species within a mixed soil sample at the same time, is the associated soil descriptor. However, the latter has to be applied only to fungi and bacteria, and only one of the additional nine soil descriptors relating to larger organisms, such as earthworms and springtails, needs to be selected. This stands in stark contrast to the findings that soils’ macrofauna are considered better bioindicators due to their greater sensitivity to environmental stressors, such as pesticides (FAO et al. 2020).

### Policy Assessment

*Lacking target stringency* is the major governance problem of the SML’s effect on reduced pesticide pollution risks and its alignment with biodiversity and soil health goals. In contrast to soil descriptors for heavy metals or organic pollutants listed under “soil contamination” in Annex I Part B SML, Member States do not have to set sustainable target or operational trigger values. Thus, an obligation to provide support and specific advice to land owners to e.g., reduce pesticide quantities or toxicity is not triggered (Art. 11 SML). In fact, with regard to pesticides, the SML merely establishes a data collection system without any immediate legal consequences. The Directive generates knowledge, and whilst the collected data could serve as a basis for future regulatory measures, such as those planned as part of the SML’s revision in 2033 (Art. 25 SML), this long time-span will aggravate the problem of lacking target stringency even further.

The SML merely creates administrative transparency but omits the necessary integration with chemicals legislation to prevent further (pesticide) releases ﻿– until binding criteria for the soil descriptors listed in Annex I Part C SML might be decided upon during the 2033 revision (Art. 25(1)(f)(ii) SML). Clearly, the emphasis of the SML is on gathering data on soil contaminants that could pose a risk to human health and the environment, including pesticides (recital 51 SML), instead of demanding concrete actions on the ground. This reveals a sharp gap between evidence gathered and regulatory response.

Having said that, even if pesticides were categorized under Annex I Part B SML, the shortcomings of the current German soil protection policies could not be remedied. Still, in that case, Member States have to establish their own, *legally non-binding* maximum values and operational trigger values for each soil descriptor. If operational trigger values are exceeded, support shall be granted for landowners, including for example information on suitable measures and practices to achieve healthy soils (Art. 11 SML). In contrast to the support provision, the initially proposed sustainable soil management practices for all managed soils under Art. 11 of the SML proposal (European Commission 2023) are no longer included in the Directive. These binding practices could have counterbalanced the lacking *legal clarity* of the good professional practice in the German soil protection legislation and pesticide use policies (sections “Federal and State Soil Protection Law” and “Pesticide Use policies”) but are now absent.

#### Federal Nature Conservation Law

The Federal Nature Conservation Law (BNatSchG) aims to protect the environment in the long-term, which includes the protection of the biological diversity and the productivity, functionality and regenerative capacity of nature (§ 1 (1) Nos. 1, 2 BNatschG). To this end, soils have to be preserved so that they can fulfill their function in the environment (§ 1(3) No. 2 BNatschG).

§ 15 BNatschG is based on the “polluter pays principle” which is another fundamental principle of environmental law (OECD 2025). According to § 15 BNatschG, interventions in nature should be avoided wherever possible, and compensatory or replacement measures be taken for unavoidable interventions (so-called “intervention regulation”). Interventions in nature are defined as changes to the form or use of land, or changes to the groundwater level (§ 14 (1) BNatschG). However, according to the prevailing legal opinion, the activities of daily agricultural practice and in particular the application of fertilizers or pesticides, are not considered an intervention in nature (Federal Administrative Court 2016).

Moreover, according to § 14 (2) BNatschG, agricultural land use does not constitute an intervention in nature if the objectives of nature and landscape conservation are considered. This is the case if the “good professional practice” of the soil protection law (section “Federal and State Soil Protection Law”) and § 5 BNatschG are complied with. According to § 5 (2) BNatschG, agricultural practices should be undertaken in a way that is compatible with nature and the landscape. Besides that, fertilizers and pesticides have to be used in accordance with agricultural law, and must be documented. Land use must be site-adapted, preserve natural soil fertility and must not exceed what is necessary to achieve a sustainable yield. Having said that, in 2016, the Federal Administrative Court (BVerwG) ruled that Section 5(2) BNatSchG does not contain binding requirements or prohibitions, but only non-binding guidelines for agricultural operations due to lacking precision (BVerwG, judgment of Sept. 1, 2016—4 C 4.15). Therefore, the reference to good professional practice runs empty, and § 14(2) BNatSchG remains an exeption clause based on the presumption that agricultural land use generally does not conflict with the goals of nature conservation, granting preferential treatment.

Having said that, within or near Natura 2000 sites, an impact assessment according to § 13 BNatschG must be conducted for agricultural land-use measures ﻿– that would otherwise not require an authorisation ﻿– if these measures could have a significant adverse effect on the Natura 2000 site and its specific conservation objectives ﻿– either individually or in conjunction with other plans and projects (ECJ, Judgment of 7 November 2018–C-293/17 and C-294/17). This impact assessment incorporates substance inputs, such as pesticides, as these have direct and indirect effects on wild species and habitats (ECJ, Judgment of 7 November 2018—C-293/17 and C-294/17, paras. 59–73). Besides that, activities like the application of pesticides need not take place within the area itself, as adverse effects arising from outside the area through long-range transport or long-range effects are also subject to assessment (ECJ, Judgment of 24 November 2011—C-404/09, paras. 146 et seq., 166 et seq.; Judgment of 13 December 2007—C-418/04, para. 256 et seq.).

In addition to these impact assessment obligations, a full environmental impact assessment must be conducted if significant adverse effects on the conservation or restoration of favorable conservation statuses cannot be ruled out with certainty (ECJ, Judgment of 7 November 2018—C-461/17). The corresponding risk must be assessed on the basis of the area’s specific characteristics and environmental conditions (ECJ, Judgment of 7 November 2018—C-293/17 and C-294/17, para. 93, 109) and according to the Court of Justice, the only measures exempt from the assessment requirement are those that were in place before the Habitats Directive (HD), (OJEC 1992) came into force in 1992 and have not changed since then (ECJ, Judgment of 7 November 2018—C-293/17 and C-294/17, paras. 74–86; Judgment of 14 January 2010—C-226/08, paras. 47–51). However, since most pesticides were authorised later, pesticide application is outside the scope of this exeption. Nevertheless, the “no deterioration” principle under Article 6(2) HD would apply in these cases. The principle requires a similarly high level of protection and obliges Member States to take preventive and repressive measures to avoid deterioration (ECJ, Judgment of 7 November 2018–C-293/17 and C-294/17, para. 85, 134; Judgment of 14 January 2016 – C-399/14, para. 52). Consequently, the use of pesticides has to be prohibited by Member States if there is any possibility that such use could adversely affect the Natura 2000 site. This applies at least if there is no exception under Article 6(4) HD, such as the absence of alternatives or possible compensatory measures (Möckel et al. 2021b, p. 21).

### Policy Assessment

In line with the findings related to the Federal Soil Protection Law in section “Federal and State Soil Protection Law”, *lacking legal clarity* and *enforcement deficits* reinforce each other and fail to induce reduced pesticide pollution risks (quantity and toxicity). While the overarching principles of § 5 BNatschG imply that pesticides have to be used based on soil properties and the sensitivity of the location rather than the maximum achievable yield, it is once again not possible to *enforce* this interpretation or issue binding actions. The vague wording and thus the *lack of legal clarity* also weaken the good professional practice as established in § 5 (2) BNatschG. The practices only include guiding principles for agricultural land use. Concrete and enforceable foundations are missing. Thus, the privileged status of agricultural land use can also not be captured by nature conservation law and remains in place. This stands in sharp contrast to the potential damage pesticide use can create for biodiversity and soil health and is neither in line with the precautionary nor the polluter pays principle. As such, the German nature conservation law outside Natura 2000 areas does not sufficiently contribute to reducing pesticide pollution risks in the use-case area and thus to biodiversity and soil health goals.

Having said that, a different conclusion is drawn for Natura 2000 areas and their vicinity. In these areas, potentially harmful pesticide use that can alter the site-specific conservation objectives has to be prohibited by Member States such as Germany. To this end, a detailed assessment is necessary, and enforcement mechanisms are established under EU nature conservation law. The governance problems described above no longer exist in these cases.

## Discussion

### Summary of Qualitative Governance Analysis

The aim of this article is to assess the extent to which German pesticide policies contribute sufficiently to reduced pesticide pollution risks and thus the goals of the CBD and SML. To this end, the SOILPROM use-case in Jülich serves as reference area for the analysis. The analysis shows that even though single measures, such as certain areal policies, contribute to reduced pesticide pollution risks, the steering effect of most measures is limited by several partly overlapping governance problems as shown in Table 3 below.

**Table 3:** Summary of the qualitative governance analysis

	Instruments on pesticide pollution risks	Typical governance problems
Pesticide use policies	Contains limited measures which can reduce pesticide quantities and toxicity in soils such as e.g., the obligation to obtain a certificate.	Steering effect substantially limited by lacking control and enforcement mechanisms as well as lacking target stringency, i.e., policy instruments fail to induce behavior change.
Pesticide spatial policies	Contains some measures which can reduce pesticide use and thus risk such as the establishment of water protection areas.	﻿Steering effect predominantely limited by lacking control and enforcement mechanisms, as well as insufficient transparency in the exeption regime.
Soil protection and nature conservation policies	Contains limited measures which aim to minimize pesticide inputs in soils so that soil toxicity risks are avoided, mainly based on the precautionary principle.	﻿Steering effect substantially limited by lacking target stringency, non-applicability due to subsidiarity (German Soil Protection legislation), and single focus on monitoring (SML). Suffers additionally from lacking legal clarity regarding the “good professional practice” and thus, no implementation and enforcement is possible.

### Policy Proposals to Reduce Pesticide Risks

Overall, identifying the direct steering effect of pesticide policies in the EU and Germany is challenging as data and monitoring remain insufficient (Bozzini 2017; European Court of Auditors 2020; Möhring et al. 2020) and simple indicators such as pesticide sales are unable to capture hidden impacts on pesticide use such as the weather (Nielsen et al. 2023). To make matters more difficult, the drivers of ecosystem degradation are numerous and the exact impacts of each driver and their potential interaction in an ecosystem are challenging to pinpoint (Wirth et al. 2025).

**Fig. 2:**
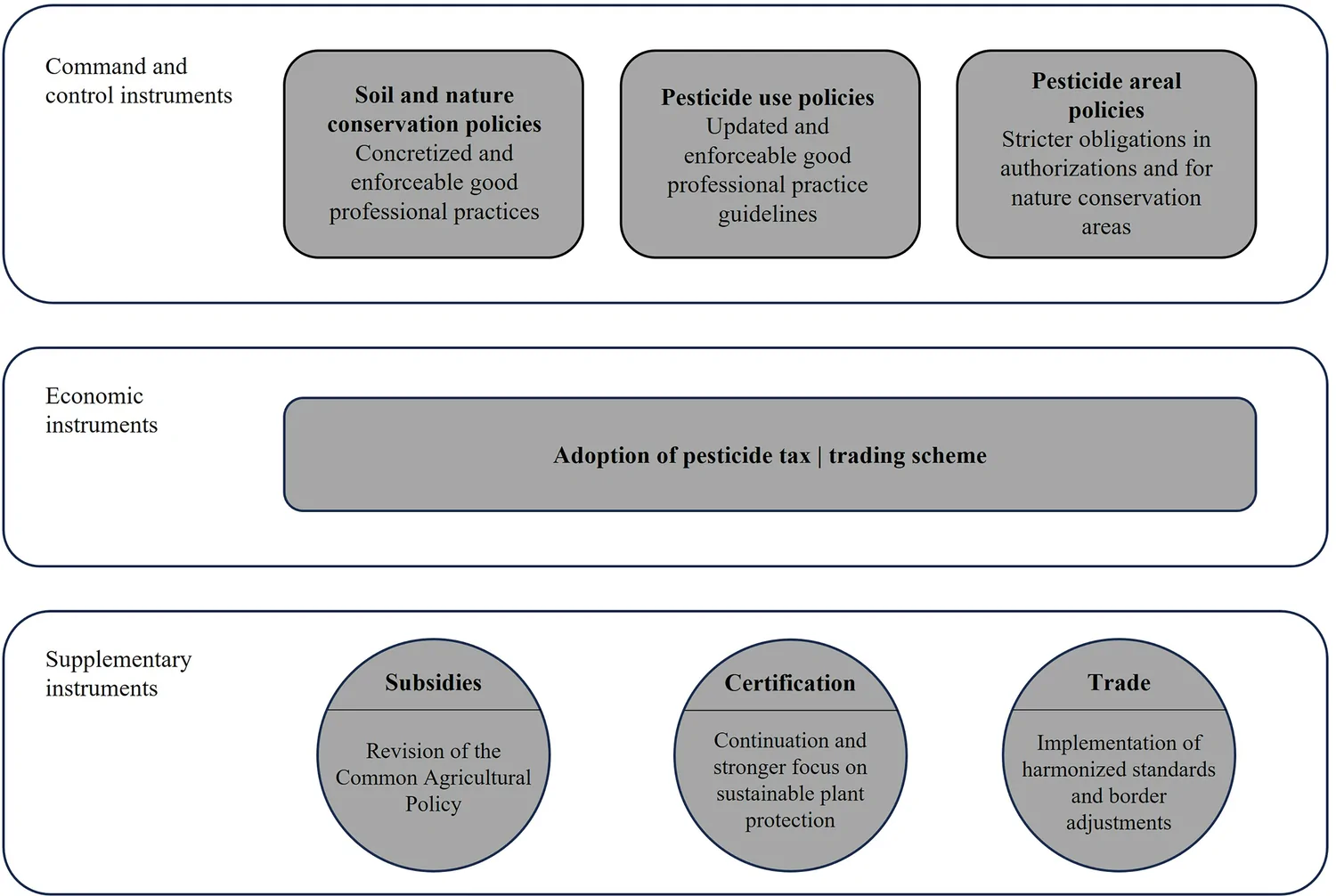
Summary of pesticide policy proposals (own figure)

To respond to uncertainty and complexity, policy has different answers. First of all, on a very fundamental level, human rights require that the preconditions for freedom such as health and ecosystem integrity must be preserved (Ekardt 2024). Besides that, legally binding policy goals such as the CBD and SML require an overall preservation and restoration of the environment, including soils. Lastly, the Treaty on the Functioning of the European Union incorporates the “polluter-pays-principle” and the “precautionary principle” which demand that those responsible for pollution have to bear the costs for it and that environmental risks have to be minimized preventively (Art. 191 (2) TFEU). Hence, given that and the fact that (soil) ecosystems in Germany and the EU continue to be under substantial pressure (European Commission. Joint Research Center and European Environment Agency 2024; Prăvălie et al. 2024; Wirth et al. 2025), effective policy is needed to address the drivers of (soil) degradation, including pesticide pollution. However, the present analysis finds that German policies largely fail to reduce pesticide pollution risks in the environment and are thus not in line with the goals of the CBD and SML, due to the identified governance problems. Against this background, the following policy proposals seem useful (Fig. 2):

#### Binding and Enforceable Pesticide Use Requirements

One option to increase the effectiveness of pesticide use requirements is to revise the good professional practice guidelines for pesticide use at the federal level. The guidelines would require (1) an update so that they reflect the current state of knowledge and become more precise, (2) a binding character by e.g., transforming them into a binding ordinance (Pitschel 2021, p. 660), (3) a comprehensive control system and (4) clearly defined central legal terms. Möckel (2017) furthermore suggests that states such as NRW could supplement these guidelines by defining clear and quantifiable minimum requirements which better reflect local circumstances. Another option is to strengthen the penalties which are e.g., established if pesticide users breach the use obligations as required in the authorization. Overall, these amendments would address problems of measurement and monitoring (“complexity issue”), control and enforcement issues and reduce lacking legal clarity.

However, even if these amendments were adopted, pesticide use requirements will continue to suffer from substantial control and enforcement problems because the number of pesticide users in agriculture is high. In Germany, 255.000 farms manage agricultural land (33.570 in NRW) and 85% of the land is managed conventionally and thus principally subject to pesticide risks (Federal Ministry of Agriculture, Food and Regional Identity 2025b; Statistic. NRW 2026). It seems very unlikely that any enforcement and control system could effectively ensure that rules are complied with by all farmers and on all land. As such, amendments to pesticide spatial measures seem to be necessary.

#### Stronger Spatial Provisions

Stronger use could be made of spatial obligations in the authorizations. For instance, authorizations could require pesticide users to devote land to pesticide-free areas or other compensatory measures (Borwieck 2017). This could contribute to the enforcement to use pesticides only on designated areas and to reduce control problems by less-control-demanding approaches. And whilst these two obligations could be supported by the subsidies of the Common Agricultural Policy or through tax revenues (see below), an open question remains as to how this would or would not counteract the “polluter pays principle”. Aside from that, stronger protective measures for nature conservation areas appear useful in order to increase target stringency to use pesticides only on designated areas. One promising option is the creation of buffer zones between conservation areas and non-conservation areas as researchers have identified pesticide residues in insects in nature conservation areas that are close to agricultural land (Brühl et al. 2021; Sinclair et al. 2025). These buffer zones would also contribute to the obligations of the Nature Restoration Regulation which requires terrestrial ecosystem restoration. Above all, monitoring and regulatory exceptions must be more clearly enforced, transparency enhanced and access to information improved.

Still, while all of these amendments are needed to reduce pesticide pollution risks in the use-case area, they will not fully address or even create new governance problems and remain highly complex. This is because as small-scale instruments, command and control measures are mostly unable to comprehensively address a complex environmental issue such as pesticide pollution (Costanza et al. 2015; Cato 2020).

#### Concretize Good Professional Practice in Soil Protection Policies

In order to reduce pesticide pollution risks and implement preventive soil protection, the good professional practice of land management should be concretized. To this end, standard examples for basic requirements and minimum ecological production standards, including pesticide use, are needed. Furthermore, clear and detailed requirements are crucial to generate enforceable and sanctionable law that goes beyond general principles and objectives. In doing so, the issues of lacking legal clarity and insufficient enforcement can be addressed to some extent. The requirements for good professional practice should therefore (1) be formulated in a comprehensible, consistent, practical and legally binding nature, (2) specify concrete qualitative or quantitative requirements for agricultural land use, (3) contain only limited and clearly defined discretionary powers for authorities (Möckel et al. 2014) while (4) allowing for sufficient scope for uncertainties in land management and (5) following the principle of proportionality. While the latter would not allow a general prohibition of chemical pesticides, an obligation to first utilize natural plant protection methods or to diversify production in favor of integrated pesticide management, and to only use (certain) chemical plant protection products as last resort, would be possible (see also Art. 14 Directive 2009/128/EC).

A specification of the good professional practice could be either included in the national or EU soil protection legislation. The EU level would be preferable as this option prevents shifting effects between Member States, as well as competitive disadvantages. Having said that, enhancing the effectiveness of the SML requires closing the gap between data generation on pesticide pollution in soils and legal action. Besides that, binding obligations for pesticide use should be enshrined. A possibility to do so is the evaluation of the Directive’s effectiveness in 2033.

Finally, competent authorities must have the power to issue preventive prohibitions on certain actions or to impose sanctions for non-compliance with the good professional practice. In addition, a clause in the BBodSchG is required which stipulates that the *lex specialis* only takes precedence if it complies with the provisions of the BBodSchG and must then be reviewed accordingly and adapted by a certain deadline. In doing so, the reference in the BNatSchG to § 17 BBodSchG would no longer be meaningless and the “precautionary principle” in soil protection no longer be undermined.

Having said all this, neither a revision of the SML nor the federal soil or nature conservation laws with enforceable rules for good professional practice in land use seem to be a realistic policy option presently. A second-best alternative is therefore to start with a revision of the *lex specialis* to strengthen pesticides regulation to reduce pesticide pollution risks in the use-case area and beyond (see sections above).

#### Adoption of Economic Policy Instruments

Previous research shows that economic instruments are suitable to address (pesticide) pollution comprehensively and thereby circumvent some governance problems of command and control instruments (Ekardt et al. 2024; Finger and Pedersen 2025). Against this background, we propose that German command and control instruments are supplemented by an economic instrument such as a tax or trading scheme for pesticides (Fig. 2).

While a flat tax which imposes a fixed rate per pesticide unit is easy to implement, a differentiated tax rate according to environmental impacts is more effective to reduce pesticide pollution risks in the environment. This is because if a uniform tax level was applied to all pesticides, pesticides users could be incentivized to switch from using large quantities of less risky pesticides to using less quantities of risky pesticides to reduce tax payments. In adopting a differentiated tax rate, these shifting effects from less toxic to more toxic taxes are avoided. A tax furthermore enforces the “polluter pays principle” and tax revenues could be used to fund compensation measures and/or the provisions of public goods by the agricultural sector (Finger et al. 2017; Skevas 2020; Nielsen et al. 2023).

An alternative to a pesticide tax are certificate trading systems – although less research has been conducted on these instruments. Dehler (2023) for instance outlines a *license scheme* which provides farmers with tradable pesticide application rights per land unit. To apply pesticides, farmers have to own a certain amount of pesticide application rights. A public authority allocates these rights to farmers each year. To reduce overall pesticide application, fewer rights are allocated to farmers in consecutive years. Farmers thus have to decide whether to reduce pesticide application on their land or buy pesticide application rights on the market. Dehler (2023) thus develops a downstream trading system. Upstream systems are an alternative. An upstream trading system targets producers or traders of pesticides – rather than users. Producers or traders have to own a certain number of allowances to sell or trade pesticides. The price of the allowance would indirectly be paid by pesticide users as producers or traders would pass the allowance cost on to customers (Ekardt et al. 2024; Gawel and Möckel 2025). While both approaches implement the “polluter pays principle”, the administrative and control burden of a downstream approach appears high while an upstream approach requires additional spatial policies to avoid pesticide hot-spots and shifting effects. This highlights that a policy mix of command and control instruments *and* economic instruments is needed to effectively govern pesticide pollution risks.

### Moving beyond Pesticide Policy

A comprehensive agricultural policy reform is needed if pesticide policies are to reduce pesticide pollution risks, induce sustainable agricultural practices and effectively contribute to biodiversity and soil health goals. Within this reform, pesticide policy will have to build on a puzzle of different policy instruments (see above and Finger et al. 2017; Lee et al. 2019; Skevas 2020), including a revision of the subsidies of the EU Common Agricultural Policy (CAP). However, despite repeated calls to align CAP subsidies with the provision of public goods, the proposal for the future CAP will not induce the changes needed to limit the negative impacts of agriculture on the environment (Cuadros-Casanova et al. 2023; Kortleve et al. 2024; Heyl et al. 2026).

The comprehensive transformation furthermore must not stop with pesticide policies. Instead, chemicals overall need to be governed more effectively in order to achieve biodiversity and soil health goals. Researchers have for instance found out that glyphosate in water originates not only from agricultural pesticide application but from laundry product degradation (Schwientek et al. 2024). Besides that, pesticide application can introduce PFAS into soils, which are highly persistent and bio-accumulative chemicals and negatively impact human health (Donley et al. 2024; Joerss et al. 2024). Thus, a transformation of chemicals policy is also needed.

Having said that, the comprehensive transformation must not stop at the German and EU border. Instead, global solutions are needed to avoid shifting (“leakage”) effects. To this end, harmonized food production standards or border adjustments are needed (Handford et al. 2015; Bozzini 2017; Li and Jennings 2017; Fuchs et al. 2020; Ekardt et al. 2024). The adoption of these standards may require support for economically weak countries and does not preclude the necessity to have strong local measures (Lee et al. 2019).

Overall, the results of this article show that there is no one obvious policy response for pesticide pollution risks but a plethora of policy options which together might be able to effectively contribute to biodiversity protection and soil health goals in the use-case area and beyond.

However, policy ambition generally seems to be low – both in Germany and the EU. At EU level, the Commission withdrew an ambitious pesticide regulation in 2024 (European Commission 2024) and the EU Parliament discusses weakening pesticide approval provisions (ENVI and AGRI 2026). Germany failed to update its National Action Plan on the Sustainable Use of Pesticides and withdrew funding from an even weaker Future Program Plant Protection (German Bundestag 2025). The program aimed to strengthen integrated pest management by updating the good professional practice guidelines, but an update never appeared. Moreover, the government in NRW in 2022 announced that it would develop a “Future Program Modern Agriculture” and a strategy to reduce plant protection products but neither has so far been published (CDU NRW and Bündnis 90—Die Grünen 2022). Hence, despite the urgency, policy change appears very challenging.

To conclude this article, we would like to point out that this study has limitations:

1. Scope of the analysis: the analysis only provides a snapshot of the applicable pesticide policies in Germany. Other measures in e.g., pesticide policy, water protection policy and agricultural policy also impact agricultural production and pesticide pollution risks.  Besides that, the analysis does not differentiate between herbicides, pesticides and fungicides. This analysis thus does not provide a complete assessment of measures influencing pesticide risks and governance problems may have been overlooked. Future research could build on and extent the present analysis to provide a more comprehensive and differentiated assessment.
2. Methodical limitations: the qualitative governance analysis assumes a more or less linear relationship between existing policies, governance problems and more effective policy proposals. In doing so, the analysis fails to capture the complextiy of policy change. Policy making is shaped by numerous factors including political negotiation, competing interests and institutional constraints. Some policy recommendations may therefore be difficult to implement in practice and are thus not straightforward or immediately implementable solutions. Future governance analyses could address lacking complexity by e.g., incorporating stakeholder perspectives, network and narrative analyses, political economy constrains and experiences from past policy reforms. These considerations could also shed some light onto why policy progress is staggering and how to implement effective measures and how to implement effective measures.
3. Use-case incorporation: the governance analysis fails to incorporate local circumstances and perspectives in detail. Consequently, results may not fully reflect local implementation challenges and should therefore be interpreted with caution. A closer and more targeted investigation into the environmental and farming conditions in the use-case area could have enabled a more targeted and localized policy assessment and future research could even more closely collaborate with e.g., stakeholders and local policy makers.

## Conclusions

Pesticide pollution in soils is a pressing environmental issue that needs to be addressed urgently. The results of this analysis indicate however that current pesticide policy in Germany suffers from multiple governance problems which undermine the effectiveness of these measures in reducing pesticide pollution risks. They are thus not in line with the legally binding policy goals of the Convention on Biological Diversity and Soil Monitoring Law. To effectively govern pesticide pollution risks, a policy mix is needed. This includes new economic and revised command and control instruments, targeted subsidies, training and advice. However, current policy developments point in a different direction.

## Data Availability

No datasets were generated or analysed during the current study.

## Footnotes

1 Number of pesticide applications in relation to the authorised application rate and the cultivated area.

2 While laws are adopted in Parliament, ordinances are adopted by the Federal or State Government, a Federal or State Ministry or an authority subordinate to or under the supervision of a State Ministry. Ordinances usually specify legal requirements or determine further obligations and restrictions. The authorisation to issue specific ordinances is provided in the relevant law. Administrative regulations and enactments are adopted by the same authorities. They serve the guidance of the administration.

3 This rule appears to be similar to the “intervention regulation” in Federal Nature Conservation Law (see section “Federal and State Soil Protection Law”).
